# Impact of single nucleotide polymorphisms of immunomodulatory factors on treatment response and prognosis in acute myeloid leukemia

**DOI:** 10.3389/fimmu.2025.1571332

**Published:** 2025-03-31

**Authors:** Mingying Li, Tao Sun, Mengyuan Chang, Tingting Liu, Lei Feng, Di Zhang, Yuyan Wu, Yuechan Ma, Huixian Ma, Guangqiang Meng, Chunyan Ji, Jingjing Ye

**Affiliations:** ^1^ Department of Hematology, Qilu Hospital of Shandong University, Cheeloo College of Medicine, Shandong University, Jinan, Shandong, China; ^2^ Shandong Key Laboratory of Hematological Diseases and Immune Microenvironment, Qilu Hospital of Shandong University, Jinan, Shandong, China; ^3^ Laboratory of Cryomedicine, Qilu Hospital of Shandong University, Jinan, Shandong, China

**Keywords:** AML, immunoregulatory factors, treatment response, prognosis, SNPs

## Abstract

**Background:**

Acute myeloid leukemia (AML) is a hematologic malignancy characterized by poor overall survival (OS). The impaired function, altered phenotype, and abnormal distribution of T cells create an immunosuppressive microenvironment in AML, affecting the efficacy of chemotherapy. Studies have shown that differentiated monocyte-like AML cells can express various immunomodulatory factors, resulting in T cell phenotypic changes and the development of an immunosuppressive AML microenvironment.

**Methods:**

Seven single nucleotide polymorphisms (SNPs) of four immunomodulatory factors—HMOX1, TXNIP, TNSF10/TRAIL, and TNFAIP2—were selected and analyzed in 255 non-M3 AML patients and 316 healthy controls. SNP genotyping was conducted using the MassARRAY platform. Furthermore, we analyzed the relationship between AML susceptibility, bone marrow (BM) blast percentage, clinical characteristics, treatment response, and prognosis with the selected SNPs.

**Results:**

The study indicated that HMOX1 rs2071746 and TNFAIP2 rs1132339 are associated with BM blasts at the diagnosis of AML patients. TXNIP rs7211 is associated with sensitivity to cytarabine- and anthracycline-induced chemotherapy in AML, while TXNIP rs9245 is associated with AML relapse. Moreover, TRAIL/TNFSF10 rs12488654 is associated with the overall survival of AML patients, and the AA genotype of TRAIL/TNFSF10 rs12488654 may be an independent favorable factor for AML prognosis.

**Conclusions:**

Our results on the association between AML and SNPs in HMOX1, TXNIP, TNSF10/TRAIL, and TNFAIP2 genes provide an important reference for predicting the treatment response and prognosis of AML patients.

## Background

Acute myeloid leukemia (AML) is a hematologic malignancy characterized by poor overall survival (OS) rates. The impacted function, phenotype, and distribution of T cells mediate an immunosuppressive microenvironment in AML, affecting chemotherapy treatment (1–4). Although new immunotherapeutic strategies have entered standard clinical practice for various solid cancers as well as specific hematologic tumors, including ALL, similar advancements have lagged in the treatment of AML (5).

AML is caused by multiple genetic events resulting in impaired differentiation of primitive myeloblasts. Primitive AML cells, often referred to as leukemia stem cells (LSCs), maintain the disease and exhibit stem cell properties such as self-renewal, quiescence, and resistance to therapy (6). Differentiated AML cells lack self-renewal capacity but may influence tumor biology through pathological effects on the tumor microenvironment or hematopoiesis. Van Galen, P et al. used single-cell transcriptomics and genotyping to identify differentiated AML cells with immunosuppressive properties (7). They identified differentiated monocytic-like AML cells that express a series of immunomodulatory factors, including tumor necrosis factor (TNF) pathway genes (TRAIL/TNFSF10, TNFAIP2) and interleukin (IL)-10 pathway genes (HMOX1), reactive oxygen species regulator (TXNIP), which lead to T cell phenotypic changes and develop immunosuppression (7, 8). Consistent with previous reports (9), the monocytic-like AML cells could suppress T cell activity *in vitro*, leading to changes in T cell phenotype and an immunosuppressive AML microenvironment. However, whether the expression of these immunomodulatory genes is associated with the clinical manifestations of AML needs further exploration.

Single nucleotide polymorphisms (SNPs) are the simplest form of DNA variation between individuals, accounting for a significant portion of genetic trait variation (10). NPs can influence promoter activity, mRNA stability, and the subcellular localization of mRNA or proteins, thus impacting the function of associated molecules (10). AML is a malignant tumor characterized by significant genetic heterogeneity, numerous gene mutations, and abnormal gene expression (11). According to the European Leukemia Network (ELN), mutations such as CEBPA, NPM1, and FLT3 are now included in risk stratification (12), highlighting the potential of SNPs in AML diagnosis, treatment response, and prognosis assessment. The role of immune-related SNPs in AML patients has been extensively studied (13–16). To better understand the heterogeneity in AML response, we investigated the association between SNPs in immunomodulatory factors and AML. We analyzed the associations of selected SNPs with AML susceptibility, bone marrow (BM) blasts, clinical characteristics, treatment response, and overall survival to guide prognostic stratification and treatment of AML.

## Methods

### Characteristics of study groups

To detect genetic polymorphisms, 255 *de novo* non-M3 AML patients were recruited from October, 2010 to December, 2021. All patients (139 males,116 females) with a median age of 49 (range 13 to 87) years were diagnosed according to National Comprehensive Cancer Network (NCCN) guidelines. Complete remission (CR) was defined as restoration of hematopoiesis in all three lineages in the bone marrow, with a blasts of less than 5%. AML relapse was defined as the presence of ≥5% peripheral blood leukemia cells or myeloid progenitors, or the infiltration of extramedullary leukemia cells after CR. Overall survival (OS) was defined as the time from admission to death and was reviewed at the last follow-up of surviving patients. Additionally, 316 healthy controls (117 males, 199 females) with a median age of 40 years (range 20 to 88) were recruited into the study. The research was approved by the Ethics Committee on Scientific Research of Shandong University Qilu Hospital (KYLL-202206-015-1). All studies were conducted in accordance with relevant guidelines, and all subjects signed written research consent in accordance with the Declaration of Helsinki.

### Study subjects

Isolation of genomic DNA from bone marrow mononuclear cells of AML patients and peripheral blood mononuclear cells of healthy controls using the TIANamp Blood DNA Kit (Tiangen Biotech, Beijing, China) and the standard salting-out method according to the manufacturer’s instructions. SNP genotyping was performed using Sequenom iPLEX and the MALDI-TOF-based MassARRAY platform (BGI Tech, Beijing, China), which involves a multiplex PCR reaction, a locus-specific single-base extension reaction, and MALDI-TOF spectrometry. Primers were designed using Assay Design Suite version 2.0 (Agena Bioscience, San Diego, CA, USA), available through the manufacturer’s online tools (https://www.mysequenom.com/Tools).

### SNP selection and genotyping

Four immunomodulatory genes were included: HMOX1, TXNIP, TNSF10/TRAIL and TNFAIP2. Use the NCBI dbSNP database and SNPinfo (https://snpinfo.niehs.nih.gov/) to select potentially functional SNPs. SNPs were selected if they met the following criteria: minor allele frequency (MAF) >5% in Chinese Han subjects; located in the 5’ UTR and 3’ UTR, which may affect transcriptional activity or binding ability of microRNA binding sites; and low linkage disequilibrium (R^2^<0.8) between SNPs. A total of seven SNPs were selected.

### Statistical analysis

Genotypic compliance with Hardy-Weinberg equilibrium (HWE) in controls was assessed by a χ^2^ test. Differences in demographic characteristics between AML cases and controls were assessed by χ^2^ test where appropriate. Age- and sex-adjusted ORs and 95% CIs for the association between SNPs and AML were determined by multivariate logistic regression analysis. Kaplan-Meyer curves were used to estimate OS and Cox regression analyses were used to assess prognostic factors for AML. All statistical analyses were performed using SPSS 26.0 software (SPSS Inc., Chicago, IL, USA). Statistical significance was defined as a two-tailed *p*<0.05 or a false discovery rate (FDR) *q*<0.05.

## Results

### SNPs selection and study populations

The selected SNPs are listed in **Table 1**. HWE and MAF were used for the initial screening of seven candidate SNPs. SNPs with *p*<0.05 in the HWE test (TNFAIP2 rs8126) or MAF<5% in the general population (HMOX1 rs2071747) were excluded from further analysis. Clinical characteristics of patients with AML are shown in **Table 2**.

**Table 1:** Selected genes and SNPs.

Gene	SNP	Variant	Variant allele	MAF	HWE (*p* value)
**HMOX1**	rs2071746	T>A	A	53.322784810%	0.507774711
	rs2071747*	G>C	C	4.430379747%	0.880489823
**TXNIP**	rs9245	G>T	T	24.050632911%	0.868887427
	rs7211	G>A	A	16.297468354%	0.330833196
**TRAIL/TNFSF10**	rs12488654	G>A	A	48.892405063%	0.992671198
**TNFAIP2**	rs8126*	T>C	C	14.556962025%	6.74143E-66
	rs1132339	C>G	G	49.367088608%	0.781269246

*SNPs were excluded in further analysis.

**Table 2:** Clinical features of patients with AML.

Characteristic	Cases n (%)
**Age (years, median, range)**	49 (13-87)
<60	189 (74)
≥60	66 (26)
Gender	
Male	139 (55)
Female	116 (45)
Bone marrow blast (%)	
**Median**	78
<70%	92 (36)
≥70%	163 (64)
WBC	
**Median (×10^9^/L)**	18.24
<100	209 (82)
≥100	46 (8)
PLT	
**Median (×10^9^/L)**	39
>50	105 (41)
≤50	150 (59)
HGB	
**Median (g/L)**	76
≥60	217 (85)
<60	38 (15)
Risk stratification	
Favorable	52 (20)
Intermediate	132 (52)
Adverse	71 (28)
Response after 1 cycle of cytarabine- and anthracycline- containing induction therapy*	
CR	95 (57)
No CR	73 (43)
Relapse after CR^#^	
Yes	46 (22)
No	166 (78)

*168 patients were evaluated after 1 cycle of cytarabine- and anthracycline- containing induction

^#^212 patients were treated and tracked for relapse

### Relationship between SNP and susceptibility to AML

We used three genetic models (co-dominant, dominant and recessive) to analyze the association between AML susceptibility and the selective SNPs. However, preliminary screening by the χ^2^ test or Fisher’s exact test showed no SNPs were associated with AML susceptibility (*p*>0.05, **Supplementary Table 1**). The results indicated that the selected SNPs were not closely related to the occurrence of AML.

### HMOX1 rs2071746 and TNFAIP2 rs1132339 are associated with BM blasts at diagnosis in patients

To further explore the value of these SNPs in AML, we analyzed the relationship between SNPs and the percentage of BM blasts (**Table 3**). The proportion of bone marrow blasts was used to reflect the extent of disease burden. A BM blast percentage of 70% or greater was considered high, while a percentage below 70% was considered low. Under the recessive model, HMOX1 rs2071746 was found to be related to the percentage of BM blast at diagnosis (*p*<0.05). After the adjustment of age and sex, the AA genotype seemed to be a protective factor for AML patients with a higher BM blasts (OR=0.561, 95% CI=0.318-0.990, *p*=0.046). Additionally, TNFAIP2 rs1132339 was found to be associated with the percentage of BM blast under the dominant model (*p*<0.05). After the adjustment of age and sex, the GC and GG genotypes tended to be protective factors for AML patients with a higher BM blasts at diagnosis (OR=0.508, 95% CI=0.260-0.991, *p*=0.047).

**Table 3:** Association between SNPs and the percentage of BM blast of AML patients.

Gene	SNP	Model	Genotype	Bone marrow blast<70%	Bone marrow blast≥70%	χ^2^ test *p* value	OR (95% CI)	Adjusted *p* value
HMOX1	rs2071746	Recessive	TT+TA	60	126	**0.037**		
			AA	32	37		0.561 (0.318-0.990)	**0.046**
TNFAIP2	rs1132339	Dominant	CC	14	43	**0.04**		
			GC+GG	78	120		0.508 (0.260-0.991)	**0.047**

OR, odds ratio; CI, confidence interval.

Bold values mean statistically significant.

### The relationship between peripheral blood characteristics and SNPs in AML patients at the first diagnosis

Considering that peripheral blood characteristics may be related to SNPs, we analyzed the association of selected SNPs with WBC, HGB, and PLT in AML patients using the χ^2^ test or Fisher’s exact test. We found the high WBC group consisted of patients with a WBC count equal to or greater than 100×10^9^/L, while the low WBC group included patients with a WBC count less than 100×10^9^/L. The high PLT group was defined as patients with PLT levels greater than 50×10^9^/L, while the low PLT group included patients with PLT levels less than or equal to 50×10^9^/L. The high HGB group included patients with an HGB level equal to or greater than 60 g/L, while the low HGB group included patients with an HGB level less than 60 g/L. However, no SNPs were found to be associated with any of these characteristics (*p*>0.05).

### TXNIP rs7211 is associated with sensitivity to cytarabine- and anthracycline-containing induction in AML

We analyzed the associations between SNPs and sensitivity to cytarabine- and anthracycline-containing agents. Among the 255 non-M3 AML patients analyzed, 212 received treatment and 168 underwent bone marrow cytomorphological assessment after the first course of induction with cytarabine- and anthracycline-containing regimens. Preliminary screening with the χ^2^ test or Fisher’s exact test showed that rs9245 in TXNIP under the recessive model and rs7211 under the dominant model were significantly correlated with response to chemotherapy (*p*<0.05). After adjustment of sex and age, and applying FDR correction, the GA and AA genotypes under the dominant model of rs7211 were found to be significantly related to response to chemotherapy (OR=2.101, 95% CI=1.053-4.194, *p*=0.035), with a decrease in the CR group (**Table 4**).

**Table 4:** Association between SNPs and AML induction therapy response.

Gene	SNP	Model	Genotype	CR	No CR	χ^2^ test *p* value	OR (95% CI)	Adjusted *p* value
TXNIP	rs9245	Recessive	GG+GT	84	71	**0.034**		
			TT	11	2		1.127 (0.329-3.863)	0.849
	rs7211	Dominant	GG	75	47	**0.036**		
			GA+AA	20	26		2.101 (1.053-4.194)	**0.035**

CR, Complete remission; OR, odds ratio; CI, confidence interval.

Bold values mean statistically significant.

### TXNIP rs9245 and TNFAIP2 rs1132339 are related to AML relapse

To clarify the relationship between immunosuppression-related SNPs and relapse in AML patients, we analyzed 212 treated non-M3 AML patients. Preliminary screening using the χ^2^ or Fisher’s exact test was conducted under three genetic models. As shown in **Table 5**, TXNIP rs9245 under co-dominant and recessive models, and the TNFAIP2 rs1132339 under co-dominant and dominant models were significantly associated with the AML relapse (*p*<0.05). After adjustment of sex and age and applying FDR correction, the TT genotype of TXNIP rs9245 under co-dominant model (OR=4.531, 95% CI=1.518-13.528, *p*=0.007) or under recessive model (OR=4.345, 95% CI=1.508-12.518, *p*=0.007) tended to be a risk factor for AML relapse. In contrast, after adjusting for sex and age and applying FDR correction, the co-dominant GC genotype (OR=0.244, 95% CI=0.106-0.561, *p*=0.001) and the dominant GC and GG genotypes (OR=0.324, 95% CI=0.157-0.671, *p*=0.002) of TNFAIP2 rs1132339 appeared to be protective factors against AML relapsed. These results suggest that TXNIP rs9245 and TNFAIP2 rs1132339 are related to long-term treatment response in AML patients.

**Table 5:** Association between SNPs and AML relapse.

Gene	SNP	Model	Genotype	No relapsed	Relapsed	χ^2^ test *p* value	OR (95% CI)	Adjusted *p* value
TXNIP	rs9245	Co-dominant	GG	102	23	**0.015**		
			GT	56	15		1.120 (0.537-2.336)	0.763
			TT	8	8		4.531 (1.518-13.528)	**0.007**
		Recessive	GG+GT	158	38	**0.004**		
			TT	8	8		4.345 (1.508-12.518)	**0.007**
TNFAIP2	rs1132339	Co-dominant	CC	31	18	**0.004**		
			GC	87	13		0.244 (0.106-0.561)	**0.001**
			GG	48	15		0.472 (0.202-1.102)	0.083
		Dominant	CC	31	18	**0.004**		
			GC+GG	135	28		0.324 (0.157-0.671)	**0.002**

OR, odds ratio; CI, confidence interval.

Bold values mean statistically significant.

### TRAIL/TNFSF10 rs12488654 is associated with AML overall survival

**Figure 1:**
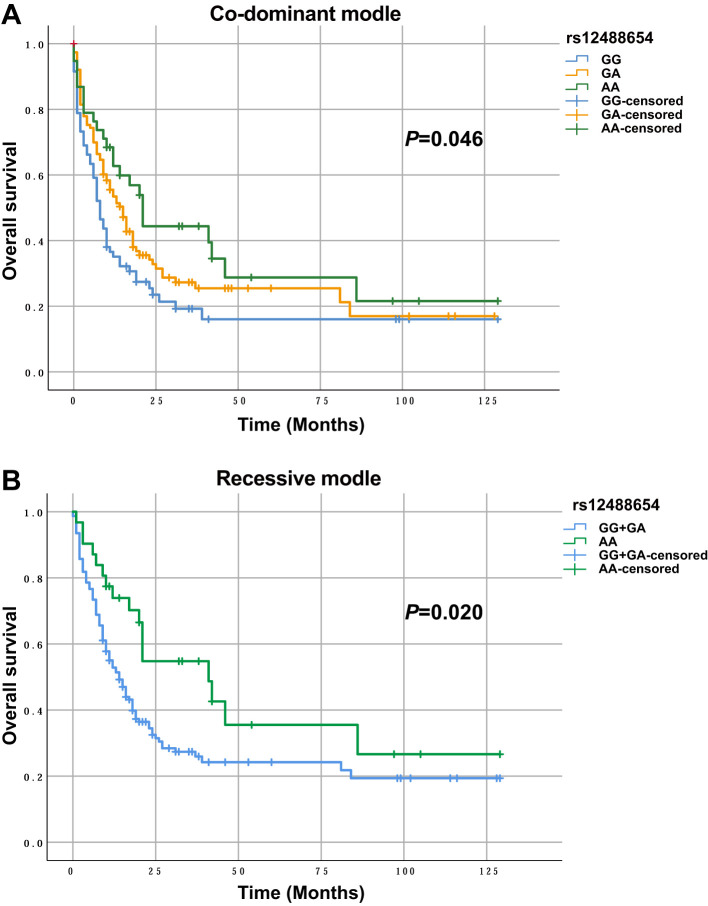
OS of AML patients with different genotypes in TRAIL/TNFSF10 rs12488654. **(A)** OS of AML patients with the GG (n=63), GA (n=112) and AA (n=37) genotypes in rs12488654. **(B)** OS of AML patients with the GG+GA (n=175) and AA (n=37) genotypes in rs12488654.

We then analyzed the relationships between various SNPs and overall survival (OS) in 212 treated AML patients. Preliminary Kaplan–Meier screening revealed that the genotype frequency of rs12488654 in TRAIL/TNFSF10 was associated with prognosis under both co-dominant and recessive models (*p*<0.05) (**Figure 1**). Other SNPs showed no significant effect on OS. For rs12488654 in TRAIL/TNFSF10, OS of the AML patients with the AA genotype was notably longer than that of patients with the GG and GA genotypes under the co-dominant and recessive models.

Patients aged 60 years or older had notably shorter OS than those younger than 60 years (*p*<0.001). Patients with a WBC of 100×10^9^/L or more had significantly shorter OS than those with a WBC below 100×10^9^/L (*p*=0.027). Patients with an HGB content less than 60 g/L had significantly shorter OS than those with an HGB content of 60 g/L or more (*p*=0.012). Patients with a PLT count below 50×10^9^/L had significantly shorter OS compared to those with 50×10^9^/L or more (*p*=0.005). Moreover, OS was significantly shorter in patients with adverse or intermediate risk stratifications compared to those with favorable risk stratifications (*p*<0.001).

### TRAIL/TNFSF10 rs12488654 is associated with the outcomes of AML patients

**Figure 2:**
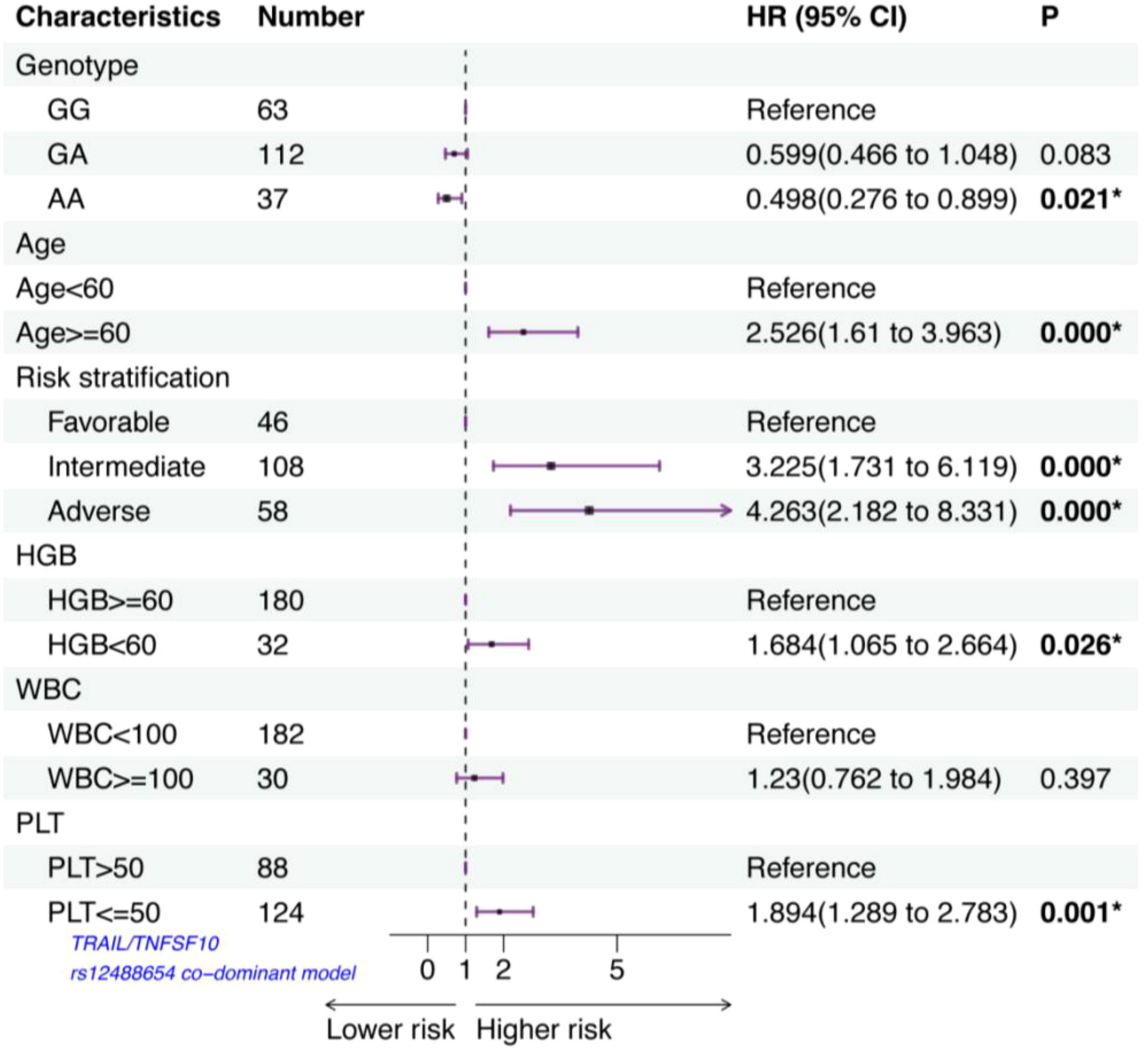
The impact of TRAIL/TNFSF10 rs12488654 on the outcomes of AML patients.

A Cox proportional hazards model with multivariate analysis for OS was used to analyze rs12488654 in TRAIL/TNFSF10, age, risk stratification, HGB, WBC and PLT count at diagnosis (**Figure 2**). In our study, age≥60 years, intermediate prognosis, adverse prognosis, HGB<60 g/L, and PLT ≤ 50×10^9^/L had an independent negative impact on OS. Patients with the AA genotype of TRAIL/TNFSF10 rs12488654 remained significantly associated with better OS (HR=0.498, 95% CI=0.276-0.899, *p*=0.021). These results demonstrate that the AA genotype of TRAIL/TNFSF10 rs12488654 is an independent favorable prognostic factor for AML patients.

## Discussion

Studies have shown that differentiated monocyte-like AML cells possess immunomodulatory functions that related to the pathogenesis of the disease (7). Monocyte-like cells overexpress genes such as TRAIL/TNFSF10, TNFAIP2, HMOX1, and TXNIP, all of which are associated with myeloid suppressor cells (7, 8, 17). Here, we discovered the association of seven SNPs across the four immunomodulatory factors in AML. Of these, five effective SNPs were further analyzed in AML patients and healthy controls. Comprehensive statistical analysis showed that none of the selected SNPs were closely related to the occurrence of AML. HMOX1 rs2071746 and TNFAIP2 rs1132339 were found to be associated with BM blasts at diagnosis in AML patients. TXNIP rs7211 was associated with sensitivity to cytarabine- and anthracycline-containing induction therapy in AML. In addition, TXNIP rs9245 and TNFAIP2 rs1132339 were associated with AML relapse. Moreover, TRAIL/TNFSF10 rs12488654 was associated with OS of AML patients, and the AA genotype of TRAIL/TNFSF10 rs12488654 may be an independent favorable factor for AML prognosis. These results suggest that the immunomodulatory genes are involved in AML treatment response and development. Even if these SNPs do not contribute to the initial development of AML, they may be associated with factors such as drug metabolism, immune response, or disease recurrence, all of which are vital for AML prognosis.

Our comprehensive study suggests that the AA genotype of TRAIL/TNFSF10 rs12488654 is an independent favorable factor with prognostic significance in AML. TRAIL/TNFSF10 is a member of the tumor necrosis factor (TNF) death ligand family that selectively initiates extrinsic apoptosis in caspase-8-dependent tumor cells without affecting normal cells (18, 19). Therefore, TRAIL/TNFSF10 is considered a promising anticancer target for selectively inducing extrinsic apoptosis in tumor cells. However, clinical trials have shown significant heterogeneity in the response of patients to TRAIL (20, 21). Notably, different types of cancer cells may have different sensitivities to TRAIL-induced apoptosis, and AML patients also show high biological and clinical heterogeneity in this regard (22). This suggests that identifying appropriate biomarkers to pre-screen patients who respond to TRAIL is an attractive option to promote the development of precision medicine for AML. However, there are no reports on the association of TRAIL/TNFSF10 SNPs with AML. Here, we found for the first time that TRAIL/TNFSF10 rs12488654 was significantly associated with AML OS. A Cox proportional hazards model with multivariate analysis for OS of rs12488654, age, risk stratification, HGB, WBC, and PLT counts at diagnosis also showed that the AA genotype of TRAIL/TNFSF10 rs12488654 may be included in the favorable stratification. This not only supplements the prognostic stratification of AML but also provides a reference for TRAIL-related targeted therapy. This valuable result deserves further confirmation in a larger AML sample study.

TNFAIP2 is another molecule in the TNF pathway implicated in the pathogenesis of AML (23). TNFAIP2 and its related genes are enriched in multiple cell differentiation pathways and upregulated during leukemic cell differentiation (23, 24). Expression of TNFAIP2 is significantly elevated in AML, especially in M4/M5 patients of French-American-British (FAB) classification. Its overexpression is an independent adverse prognostic factor affecting OS and is associated with adverse cytogenetic risk and gene mutations in AML patients (23). TNFAIP2 SNPs are associated with prognosis and risk stratification in a variety of tumors (25, 26), including squamous cell carcinoma and gastric cancer, strongly suggesting their potential role in AML. In our study, we found that TNFAIP2 rs1132339 C>G is inclined to be a protective factor associated with high BM blasts at diagnosis and AML recurrence. The impact of rs1132339 on TNFAIP2 expression and function, and its potential mechanism in AML, deserve further exploration.

Cytarabine- and anthracycline- containing regimens have been the mainstay of AML treatment for more than 50 years. The impact of genetic polymorphisms on the efficacy of induction chemotherapy in AML patients has been widely studied (27–30). In our study, rs7211 in TXNIP was found to be related to reactions induced by the first course of cytarabine- and anthracycline-containing drugs in AML, with the dominant model indicating that the GA and AA genotypes of this SNP are related to the reduction of CR rate after chemotherapy. Furthermore, TXNIP gene rs9245 was associated with AML recurrence, and the TT genotype of TXNIP rs9245 tended to be a risk factor for AML recurrence in both co-dominant and recessive models. TXNIP, a thioredoxin-interacting protein, has been reported to contribute to the growth of leukemia cells (31). TXNIP acts as a negative regulator of the key antioxidant system thioredoxin (32). Additionally, TXNIP is involved in other signaling pathways and plays a role in inflammatory responses. Lower TXNIP expression is associated with a good prognosis in AML (33). However, no study has reported the correlation between TXNIP SNP and AML. This is the first report of the relationship between TXNIP SNPs and the response to the first course of cytarabine- and anthracycline- containing induction and the long-term relapse rate of AML patients, which has important implications for using these regimens in AML treatment. However, these results require further confirmation in studies with larger AML sample sizes.

In summary, this study showed that SNPs in immunomodulatory factors might be linked to BM blasts at diagnosis, chemotherapy response, and prognosis of AML. Given the widespread accessibility of SNP genotyping assays and, pre-genotyping with rapid turnaround times can be efficiently implemented in various clinical settings using different sample types, including blood or buccal swabs. In addition, these SNPs are widely available as they are relatively common in the Chinese population. Therefore, prospective evaluation of these SNPs in clinical laboratories is entirely feasible. Our results open the door for enhancing personalized immunotherapy regimens through genomic profiling of AML patients, with the potential to extend the study cohort to better clarify the prognostic value of TRAIL/TNFSF10 rs12488654. However, the clinical sample size and study methodology used in this study have limitations. Firstly, the limited clinical sample size may limit the robustness and comprehensiveness of multivariate analysis of all variables. In addition, the exact immune regulatory molecular and cellular mechanisms of the identified SNPs deserve further study. In the future, it is necessary to repeat these results in independent cohorts and perform functional experiments to verify how these SNPs influence AML prognosis.

## Conclusion

SNPs in immunomodulatory factors may serve as genetic markers to aid in the early diagnosis of AML. HMOX1 rs2071746 and TNFAIP2 rs1132339 were found to be associated with BM blasts at diagnosis in AML patients. In addition, SNPs in immunoregulatory factors were associated with treatment response and prognosis in AML. For example, TXNIP rs7211 was associated with AML sensitivity to cytarabine- and anthracycline-containing induction therapies; TXNIP rs9245 and TNFAIP2 rs1132339 were associated with AML relapse. Furthermore, the AA genotype of TRAIL/TNFSF10 rs12488654 was associated with OS in AML patients and may be an independent favorable factor for AML prognosis that warrants attention.

## Data Availability

The original contributions presented in the study are included in the article/**Supplementary Material**. Further inquiries can be directed to the corresponding authors.

## References

[1] Mussai F De Santo C Abu-Dayyeh I Booth S Quek L McEwen-Smith RM. Acute myeloid leukemia creates an arginase-dependent immunosuppressive microenvironment. Blood. (2013) 122:749–58. doi: 10.1182/blood-2013-01-480129 PMC3731930 23733335

[2] Knaus HA Berglund S Hackl H Blackford AL Zeidner JF Montiel-Esparza R. Signatures of CD8+ T cell dysfunction in AML patients and their reversibility with response to chemotherapy. JCI Insight. (2018) 3. doi: 10.1172/jci.insight.120974 PMC6238744 30385732

[3] Li Z Philip M Ferrell PB. Alterations of T-cell-mediated immunity in acute myeloid leukemia. Oncogene. (2020) 39:3611–9. doi: 10.1038/s41388-020-1239-y PMC7234277 32127646

[4] Ozkazanc D Yoyen-Ermis D Tavukcuoglu E Buyukasik Y Esendagli G. Functional exhaustion of CD4(+) T cells induced by co-stimulatory signals from myeloid leukaemia cells. Immunology. (2016) 149:460–71. doi: 10.1111/imm.2016.149.issue-4 PMC5095494 27565576

[5] Lichtenegger FS Krupka C Haubner S Kohnke T Subklewe M. Recent developments in immunotherapy of acute myeloid leukemia. J Hematol Oncol. (2017) 10:142. doi: 10.1186/s13045-017-0505-0 28743264 PMC5526264

[6] Pollyea DA Jordan CT. Therapeutic targeting of acute myeloid leukemia stem cells. Blood. (2017) 129:1627–35. doi: 10.1182/blood-2016-10-696039 28159738

[7] van Galen P Hovestadt V Wadsworth Ii MH Hughes TK Griffin GK Battaglia S. Single-cell RNA-seq reveals AML hierarchies relevant to disease progression and immunity. Cell. (2019) 176:1265–81 e24. doi: 10.1016/j.cell.2019.01.031 30827681 PMC6515904

[8] Hartwig T Montinaro A von Karstedt S Sevko A Surinova S Chakravarthy A. The TRAIL-induced cancer secretome promotes a tumor-supportive immune microenvironment via CCR2. Mol Cell. (2017) 65:730–42 e5. doi: 10.1016/j.molcel.2017.01.021 28212753 PMC5316415

[9] Austin R Smyth MJ Lane SW. Harnessing the immune system in acute myeloid leukaemia. Crit Rev Oncol Hematol. (2016) 103:62–77. doi: 10.1016/j.critrevonc.2016.04.020 27247119

[10] Shastry BS. SNPs: impact on gene function and phenotype. Methods Mol Biol. (2009) 578:3–22. doi: 10.1007/978-1-60327-411-1_1 19768584

[11] Kumar CC. Genetic abnormalities and challenges in the treatment of acute myeloid leukemia. Genes Cancer. (2011) 2:95–107. doi: 10.1177/1947601911408076 21779483 PMC3111245

[12] Dohner H Estey EH Amadori S Appelbaum FR Buchner T Burnett AK. Diagnosis and management of acute myeloid leukemia in adults: recommendations from an international expert panel, on behalf of the European LeukemiaNet. Blood. (2010) 115:453–74. doi: 10.1182/blood-2009-07-235358 19880497

[13] Wang H Hua M Wang S Yu J Chen C Zhao X. Genetic polymorphisms of IL-18 rs1946518 and IL-1beta rs16944 are associated with prognosis and survival of acute myeloid leukemia. Inflammation Res. (2017) 66:249–58. doi: 10.1007/s00011-016-1012-4 27928589

[14] Sanchez-Maldonado JM Campa D Springer J Badiola J Niazi Y Moniz-Diez A. Host immune genetic variations influence the risk of developing acute myeloid leukaemia: results from the NuCLEAR consortium. Blood Cancer J. (2020) 10:75. doi: 10.1038/s41408-020-00341-y 32678078 PMC7366925

[15] Nursal AF Pehlivan M Sahin HH Pehlivan S. The associations of IL-6, IFN-gamma, TNF-alpha, IL-10, and TGF-beta1 functional variants with acute myeloid leukemia in Turkish patients. Genet Test Mol Biomarkers. (2016) 20:544–51. doi: 10.1089/gtmb.2016.0036 27486989

[16] Liu Q Hua M Yan S Zhang C Wang R Yang X. Immunorelated gene polymorphisms associated with acute myeloid leukemia. Clin Exp Immunol. (2020) 201:266–78. doi: 10.1111/cei.13446 PMC7419888 32349161

[17] Veglia F Perego M Gabrilovich D. Myeloid-derived suppressor cells coming of age. Nat Immunol. (2018) 19:108–19. doi: 10.1038/s41590-017-0022-x PMC5854158 29348500

[18] Montinaro A Walczak H. Harnessing TRAIL-induced cell death for cancer therapy: a long walk with thrilling discoveries. Cell Death Differ. (2023) 30:237–49. doi: 10.1038/s41418-022-01059-z PMC9950482 36195672

[19] Maji A Paul A Sarkar A Nahar S Bhowmik R Samanta A. Significance of TRAIL/Apo-2 ligand and its death receptors in apoptosis and necroptosis signalling: Implications for cancer-targeted therapeutics. Biochem Pharmacol. (2024) 221:116041. doi: 10.1016/j.bcp.2024.116041 38316367

[20] Dianat-Moghadam H Heidarifard M Mahari A Shahgolzari M Keshavarz M Nouri M. TRAIL in oncology: From recombinant TRAIL to nano- and self-targeted TRAIL-based therapies. Pharmacol Res. (2020) 155:104716. doi: 10.1016/j.phrs.2020.104716 32084560

[21] Thapa B Kc R Uludag H. TRAIL therapy and prospective developments for cancer treatment. J Control Release. (2020) 326:335–49. doi: 10.1016/j.jconrel.2020.07.013 32682900

[22] Ediriwickrema A Gentles AJ Majeti R. Single-cell genomics in AML: extending the frontiers of AML research. Blood. (2023) 141:345–55. doi: 10.1182/blood.2021014670 PMC10082362 35926108

[23] Lin MS Zhong HY Yim RL Chen QY Du HL He HQ. Pan-cancer analysis of oncogenic TNFAIP2 identifying its prognostic value and immunological function in acute myeloid leukemia. BMC Cancer. (2022) 22:1068. doi: 10.1186/s12885-022-10155-9 36243694 PMC9571470

[24] Jia L Shi Y Wen Y Li W Feng J Chen C. The roles of TNFAIP2 in cancers and infectious diseases. J Cell Mol Med. (2018) 22:5188–95. doi: 10.1111/jcmm.2018.22.issue-11 PMC6201362 30145807

[25] Zhang J Yu H Zhang Y Zhang X Zheng G Gao Y. A functional TNFAIP2 3’-UTR rs8126 genetic polymorphism contributes to risk of esophageal squamous cell carcinoma. PloS One. (2014) 9:e109318. doi: 10.1371/journal.pone.0109318 25383966 PMC4226436

[26] Guo F Xu Q Lv Z Ding HX Sun LP Zheng ZD. Correlation between TNFAIP2 gene polymorphism and prediction/prognosis for gastric cancer and its effect on TNFAIP2 protein expression. Front Oncol. (2020) 10:1127. doi: 10.3389/fonc.2020.01127 32793480 PMC7394262

[27] Megias-Vericat JE Rojas L Herrero MJ Boso V Montesinos P Moscardo F. Positive impact of ABCB1 polymorphisms in overall survival and complete remission in acute myeloid leukemia: a systematic review and meta-analysis. Pharmacogenomics J. (2016) 16:1–2. doi: 10.1038/tpj.2015.79 26503809

[28] Kim DH Park JY Sohn SK Lee NY Baek JH Jeon SB. Multidrug resistance-1 gene polymorphisms associated with treatment outcomes in *de novo* acute myeloid leukemia. Int J Cancer. (2006) 118:2195–201. doi: 10.1002/ijc.v118:9 16331627

[29] He H Yin J Li X Zhang Y Xu X Zhai M. Association of ABCB1 polymorphisms with prognostic outcomes of anthracycline and cytarabine in Chinese patients with acute myeloid leukemia. Eur J Clin Pharmacol. (2015) 71:293–302. doi: 10.1007/s00228-014-1795-6 25567217

[30] Cheong HS Koh Y Ahn KS Lee C Shin HD Yoon SS. NT5C3 polymorphisms and outcome of first induction chemotherapy in acute myeloid leukemia. Pharmacogenet Genomics. (2014) 24:436–41. doi: 10.1097/FPC.0000000000000072 25000516

[31] Erkeland SJ Palande KK Valkhof M Gits J Danen-van-Oorschot A Touw IP. The gene encoding thioredoxin-interacting protein (TXNIP) is a frequent virus integration site in virus-induced mouse leukemia and is overexpressed in a subset of AML patients. Leuk Res. (2009) 33:1367–71. doi: 10.1016/j.leukres.2009.02.027 19327827

[32] Hwang J Suh HW Jeon YH Hwang E Nguyen LT Yeom J. The structural basis for the negative regulation of thioredoxin by thioredoxin-interacting protein. Nat Commun. (2014) 5:2958. doi: 10.1038/ncomms3958 24389582 PMC3941024

[33] Chen J Hou Q Chang T Zheng J Yao C He J. Analysis of prognostic biomarker models of TXNIP/NLRP3/IL1B inflammasome pathway in patients with acute myeloid leukemia. Int J Med Sci. (2024) 21:1438–46. doi: 10.7150/ijms.96627 PMC11186430 38903927

