# Supplementary material for: Impact of single nucleotide polymorphisms of immunomodulatory factors on treatment response and prognosis in acute myeloid leukemia
Source: Front Immunol. 2025 Mar 31;16:1571332. doi: 10.3389/fimmu.2025.1571332 (PMC11994645; doi:10.3389/fimmu.2025.1571332)
Supplement: Supplementary file 1 [file DataSheet1.pdf]

Supplementary Table 1. Relationship between SNP and susceptibility to AML.

Gene	SNP	Model	Genotype	Control (n)	AML case (n)	χ^2 test *p* value
**TRAIL/ TNFSF10**	rs12488654	Co-dominant	GG	82	78	
			GA	159	129	
			AA	75	48	0.264
		Dominant	GG	82	78	
			GA+AA	234	177	0.220
		Recessive	GG+GA	241	207	
			AA	75	48	0.156
**TXNIP**	rs9245	Co-dominant	GG	184	150	
			GT	112	85	
			TT	20	20	0.722
		Dominant	GG	184	150	
			GT+TT	132	105	0.886
		Recessive	GG+GT	296	235	
			TT	20	20	0.481
	rs7211	Co-dominant	GG	225	178	
			GA	79	72	
			AA	12	5	0.333
		Dominant	GG	225	178	
			GA+AA	91	77	0.715
		Recessive	GG+GA	304	250	
			AA	12	5	0.199
**TNFAIP2**	rs1132339	Co-dominant	CC	82	57	
			CG	156	126	
			GG	78	72	0.490
		Dominant	CC	82	57	
			CG+GG	234	198	0.319
		Recessive	CC+CG	238	183	
			GG	78	72	0.338
**HMOX1**	rs2071746	Co-dominant	TT	95	76	
			TA	147	110	
			AA	74	69	0.574
		Dominant	TT	95	76	
			TA+AA	221	179	0.964
		Recessive	TT+TA	242	186	
			AA	74	69	0.318
